# A Case of Vaginal Stillbirth in the Presence of Placenta Previa at 33 Weeks of Gestation

**DOI:** 10.1155/2016/9872561

**Published:** 2016-08-07

**Authors:** Yukiko Chinen, Tadatsugu Kinjo, Hayase Nitta, Yui Kinjo, Hitoshi Masamoto, Yoichi Aoki

**Affiliations:** Department of Obstetrics and Gynecology, Graduate School of Medicine, University of the Ryukyus, Okinawa 903-0215, Japan

## Abstract

It was demonstrated that second- and third-trimester therapeutic termination of pregnancy (TOP) is feasible in cases with placenta previa. We report a 34-year-old woman with complex fetal malformations associated with placenta previa. An ultrasound examination at 21 weeks of gestation revealed fetal growth restriction (FGR) and complex fetal malformations associated with a placenta previa. After extensive information, the parents opted for careful observation. Thereafter, FGR gradually progressed and we observed arrest of end-diastolic velocity of the umbilical artery. Finally, intrauterine fetal death (IUFD) was confirmed at 33 weeks of gestation. Two days after IUFD, the patient experienced labor pain. The placenta and dead fetus weighing 961 g were vaginally delivered, and total bleeding was 270 mL. Although further studies to confirm the dynamic change of the uteroplacental blood flow are necessary to avoid the risk of maternal hemorrhage, vaginal TOP with placenta previa after feticide or IUFD would be feasible.

## 1. Introduction

While attempting therapeutic termination of pregnancy (TOP), inducing labor at second or third trimester is associated with an increased risk of maternal hemorrhage in cases with placenta previa [1]. Recently, it was demonstrated that second- and third-trimester therapeutic TOP is feasible in cases with complete placenta previa and that the incidence of maternal hemorrhage decreases when feticide is performed a few days before delivery [2–4].

Here we report a case with complex fetal malformations associated with placenta previa vaginally delivered at 33 weeks of gestation 2 days after intrauterine fetal death (IUFD).

## 2. Case Report

A 34-year-old woman (gravida 0, para 0) was referred to our hospital at 15 weeks of gestation for a suspicious cystic hygroma because of edema around a fetal head. We did not observe the edema or nuchal translucency on ultrasound examination. The patient was carefully followed up. However, an ultrasound examination at 21 weeks of gestation revealed complex fetal malformations [fetal growth restriction (FGR), oligohydramnios, cleft lip, hypoplasia of vermis cerebellum, dilatation of cisterna magna, and holoprosencephaly] associated with a placenta previa covering the internal cervical os. Trisomy 13 syndrome was highly suspected because of holoprosencephaly, cleft lip, and so on. The oligohydramnios was caused by the trisomy 13 and also by uteroplacental insufficiency.

**Figure 1:**
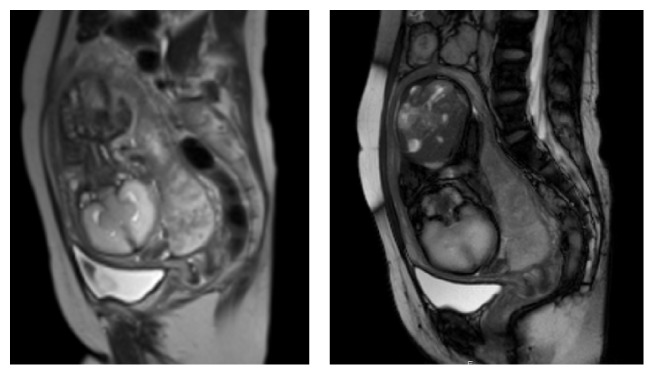
Partial placenta previa, severe oligohydramnios, and holoprosencephaly (semilobar type) by magnetic resonance imaging (MRI) at 32 weeks of gestation.

**Figure 2:**
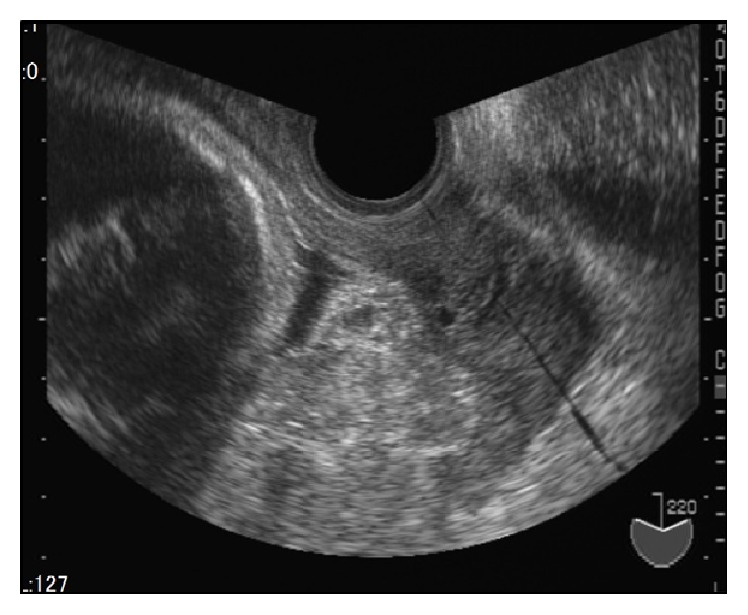
Two days after intrauterine fetal death (at 33 weeks and 4 days of gestation), the placenta previa was studded with irregular high-echo regions.

**Figure 3:**
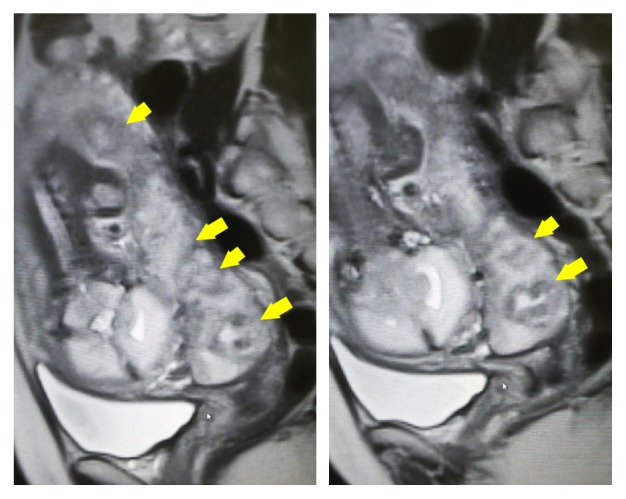
T2-weighted image showed a mixture of hyperintense and hypointense lesions (arrows) in the placenta, representing cotyledon infarction.

After being provided with extensive information on the severity of the neonatal prognosis, the parents opted for careful observation but not TOP. Thereafter, FGR (731 g, −4.5 SD at 31 weeks of gestation) gradually progressed and we observed absence of end-diastolic velocity (AEDV) of the umbilical artery.  Figure 1 shows partial placenta previa and holoprosencephaly by magnetic resonance imaging (MRI) at 32 weeks of gestation. The placenta was studded with irregular high-echo regions and a considerable reduction in placental blood supply was estimated by ultrasound examination (Figure 2) and MRI (Figure 3). Finally, IUFD was confirmed at 33 weeks and 2 days of gestation. Two days after IUFD, the patient experienced labor pain. The placenta and dead fetus weighing 961 g were vaginally delivered, and total maternal bleeding was estimated to be 270 mL.

## 3. Discussion

For placenta previa in a patient requiring second- and third-trimester TOP, a cesarean delivery was initially considered as the safest mode of delivery. However, several reports are available on successful vaginal uterine evacuation despite an increased risk of maternal bleeding. No apparent increase in abortion-related infections, postoperative transfusion requirements, hysterectomy, or other complications has been reported for second-trimester pregnancy (at 13–24 weeks of gestation) terminations in the presence of placenta previa [1]. Late midtrimester (19–24 weeks of gestation) pregnancy termination by dilatation and evacuation in the presence of placenta previa apparently did not increase maternal morbidity compared with the outcome in patients without placenta previa undergoing the same procedure [5]. Furthermore, in women with placenta previa at 12–21 weeks of gestation, there was no statistical difference in the mean intraoperative blood loss between patients with and without placenta previa, although 1 patient developed serious bleeding requiring blood transfusion [6]. However, we should still consider an increased risk of maternal hemorrhage associated with placenta previa.

It has been demonstrated that feticide before induction may reduce uteroplacental blood flow and thus reduce the risk of maternal hemorrhage associated with this procedure. A French group reported that of the 15 women in their study with complete placenta previa at an average of 22.4 weeks (range, 18–33 weeks) of gestation who underwent labor induction 9 underwent labor induction without previous feticide. Of these 9 women, 4 required blood transfusions and 1 had a hemostat hysterectomy. Only 1 of the remaining 6 patients with preinduction feticide required a transfusion. They suggested that feticide before inducing labor may reduce maternal blood loss; however, they could not substantiate this with objective evidence [2].

Thus, the Doppler ultrasound examination in cases with placenta previa feticide performed a few days before labor induction demonstrated that although a reduction in placental blood supply could be observed indirectly by an increase in resistance of the uterine arteries, it could be quantified directly using the placental vascularization index (VI) of a 3-dimensional power Doppler ultrasound. Analysis of the variation in VI after feticide indicated that a considerable reduction in placental blood supply may occur 2 days after feticide [3].

Furthermore, contrast-enhanced ultrasound was used to quantify the dynamic changes in uteroplacental blood flow before and after the interruption of fetal villus circulation resulting from feticide during a second-trimester pregnancy termination in a patient with complete placenta previa. The results suggested that placental blood flow reduction after interruption of fetal circulation was a progressive and delayed mechanism. Their interpretation is that, initially, the interruption of the fetal circulation may lead to villous edema, resulting in a subsequent decrease in spiral artery blood flow with a possible delayed progressive evolution [4]. This may be consistent with several clinical studies [2–4] demonstrating that even after feticide performed a few days before labor induction, second- or third-trimester TOP carries a substantial risk of maternal hemorrhage.

In our patient, FGR gradually progressed and we observed AEDV of the umbilical artery at 31 weeks of gestation. The placenta was studded with irregular high-echo regions which represented calcifications. The presence of preterm placental calcification is reported to be a predictor of poor uteroplacental flow and adverse pregnancy outcome by Viero et al. [7]. Furthermore, preterm placental calcification is not physiologic, but pathologic, which implies that early calcification could have a different mechanism from that of placental calcification at term, leading to risk assessment for stillbirth [8]. Calcification in pregnancies complicated by preeclampsia and FGR was related to an increasing umbilical artery pulsatility index [9] and abnormal placental appearance (e.g., placental calcification or lake) at second-trimester ultrasound scan was found to correlate with placental infarction and uteroplacental dysfunction [10]. Accordingly, a considerable reduction in placental blood supply was estimated in our patient.

Because we did not perform contrast-enhanced MRI, the reduction of placental blood supply could not be clearly shown. However, infarction with hemorrhage is depicted as diffuse/circumscribed hypointense lesion and ischemic infarction without hemorrhage is demonstrated as diffuse/circumscribed hyperintense lesion in T2-weighted image [11]. In our case, T2-weighted image showed a mixture of hyperintense and hypointense lesions representing cotyledon infarction. We estimated a considerable reduction in placental blood supply also by MRI.

Thus, total maternal blood loss was estimated to be only 270 mL. Although further studies to confirm the dynamic change of the uteroplacental blood flow are necessary to achieve a well-organized delivery and to avoid the risk of maternal hemorrhage, vaginal TOP with complete placenta previa after feticide or IUFD would be feasible.
